# The prevalence of *Cyclospora cayetanensis* in water: a systematic review and meta-analysis

**DOI:** 10.1017/S0950268821002521

**Published:** 2021-12-10

**Authors:** Thivya Naganathan, Annette O'Connor, Jan M. Sargeant, Karen Shapiro, Sarah Totton, Charlotte Winder, Amy L. Greer

**Affiliations:** 1 Department of Population Medicine, University of Guelph, Guelph ON N1G 2W1 Canada; 2 Department of Large Animal Clinical Sciences, College of Veterinary Medicine, Michigan State University, East Lansing, MI, 48824 USA; 3 Department of Pathology, Microbiology and Immunology, School of Veterinary Medicine, University of California, Davis, CA 95616 USA

**Keywords:** Cyclospora, emerging infectious disease, meta analysis, prevalence, systematic review, water

## Abstract

*Cyclospora cayetanensis* is an emerging food- and waterborne pathogen that causes cyclosporiasis, a gastrointestinal disease in humans. The parasite is endemic in tropical and subtropical regions; however, its prevalence is largely dependent on environmental factors, such as climate and rainfall patterns. The objective of this paper was to conduct a systematic review and meta-analysis to determine the prevalence of *C. cayetanensis* in water and to determine if geography, water source and other variables influence this prevalence. A literature search was performed using search terms relating to water and *C. cayetanensis* in MEDLINE^®^, CAB Direct, Food Science and Technology Abstracts, Agricola databases and Environmental Science Index. Observational studies published in English after 1979 were eligible. Screening, data extraction and risk-of-bias assessment were performed independently by two reviewers. A multi-level random-effects meta-analysis was completed to determine the prevalence of *C. cayetanensis* in water and subgroup meta-analyses were performed to explore between-study heterogeneity. The search identified 828 unique articles, and after the screening, 33 articles were included in the review. The pooled prevalence of *C. cayetanensis* in water was 6.90% [95% confidence interval (CI) 2.25%–13.05%, *I*^2^ = 84.38%]. Subgroup meta-analyses revealed significant differences in the prevalence between continents. Additionally, laboratory methods between studies were highly variable and these findings highlight the need for further environmental research on *C. cayetanensis* in water using detection methods that include PCR and sequencing to accurately identify the organism. The results of this study can be used to help assess the risk of waterborne cyclosporiasis.

## Introduction

### Rationale

Globally, waterborne pathogens are a major cause of morbidity and mortality. It is estimated that contaminated water is responsible for 4.0% of deaths and 5.7% of disease burden (measured in disability-adjusted life years) around the world [1]. Many waterborne pathogens cause diarrhoeal diseases, which in 2016 were estimated to be responsible for 1.6 million deaths worldwide [2]. Protozoan parasites are of particular concern, as more than 58 million cases of diarrhoea in children detected per year are attributed to these pathogens [3].

*Cyclospora cayetanensis* is an emerging food- and waterborne coccidian parasite that causes the gastrointestinal disease cyclosporiasis in humans [4]. The first published case of human *Cyclospora* infection dates back to 1979 [5]; however, the parasite was not fully characterised and named until 1994 [6]. *C. cayetanensis* is the only human pathogenic species of *Cyclospora*. *Cyclospora* is difficult to speciate at the microscopic level, and molecular methods combined with sequencing are the most accurate way to differentiate *C. cayetanensis* from other species of the same genus [7, 8].

*Cyclospora cayetanensis* is transmitted through the faecal-oral route. For the parasite to become pathogenic it must first be shed through faecal matter. At this stage, under laboratory conditions, unsporulated oocysts require 7–15 days at temperatures between 23–27°C before becoming sporulated and infectious to humans [9]. Common symptoms of cyclosporiasis include diarrhoea, weight loss, abdominal discomfort and nausea [10]. In areas where *C. cayetanensis* is endemic, asymptomatic infection is more frequent and younger children and the elderly experience more severe symptoms [11]. Also, people living with HIV/AIDS are at higher risk of experiencing severe illness from cyclosporiasis [12]. The majority of people who have healthy immune systems usually recover without treatment, but symptoms may last from a few days to a month or longer [13].

Cyclosporiasis is most commonly reported in people living in or travelling to subtropical regions, such as Guatemala and Mexico, where *C. cayetanensis* can be naturally found in the environment [11, 14, 15]. However, sporadic and non-travel related outbreaks of *C. cayetanensis* infections are becoming more common in industrialised countries, including the United States and Canada, where *C. cayetanensis* is not known to be present in the environment. These cases have been mostly attributed to contaminated produce imported from regions where *C. cayetanensis* is endemic [16, 17]. However, the U.S. Food and Drug Administration recently reported *C. cayetanensis* contamination in domestically grown fresh produce suggesting possible endemicity in the United States [18].

Water is an important route of transmission and both sporadic and endemic cases have been traced back to contaminated water [19]. To better quantify the risk of cyclosporiasis infection, it is essential to determine the prevalence of C. *cayetanensis* in water. The prevalence of *C. cayetanensis* can depend on factors such as geographical region, season and sources of water; however, the extent of this variation is unknown.

There are two general approaches that are used to detect *C. cayetanensis* in water. These include microscopy (light and fluorescent) and molecular detection via polymerase chain reaction (PCR, e.g. real-time or qPCR and conventional single reaction or nested PCR) [8]. Microscopy is the traditional method of detecting *C. cayetanensis* and uses morphology or autofluorescence of oocysts to identify the parasite. However, this method has poor sensitivity and specificity [20]. PCR methods are frequently used to more accurately identify and speciate *Cyclospora* spp., but the results may not be sufficient to speciate the parasite. Therefore, sequencing has been used in some studies to definitively identify PCR amplification products as *C. cayetanensis* [8].

*Cyclospora cayetanensis*, like other waterborne pathogens, can be present in water at very low concentrations and samples can contain different contaminants such as bacteria, insects, faeces and other free-living organisms (e.g. algae, bacteria) which can make the parasite difficult to detect [21]. Concentration techniques such as filtration, sedimentation, or flocculation and centrifugation, are used to overcome these challenges and increase the likelihood of detecting *C. cayetanensis* in different types of water [21].

The objective of this paper was to conduct a systematic review and meta-analysis to determine the prevalence of C. cayetanensis in water and to explore whether various factors, such as geography and water source influence this prevalence. This manuscript was prepared using the guidelines outlined by the Preferred Reporting Items for Systematic Reviews and Meta-Analysis (PRISMA) [22]

## Methods

### Protocol

The *a priori* protocol for this review is available in the University of Guelph's repository and electronically published with Systematic Reviews for Animals and Food (SYREAF). The protocol can be found at https://atrium.lib.uoguelph.ca/xmlui/bitstream/handle/10214/18106/CyclosporaSysReviewProt_FinalSigned_20200725.pdf?sequence=1&isAllowed=y and http://www.syreaf.org/protocol/.

### Eligibility criteria

The population of interest was sources of water that have been tested for *C. cayetanensis* and the primary outcome was the prevalence of *C. cayetanensis*. Since many studies have not included sequence analysis to identify the parasite to the species level, prevalence information on *Cyclospora* spp. was also collected if the primary stated objective of the study was to investigate *C. cayetanensis* or if the water type (e.g. sewage) investigated was likely contaminated by human faecal matter.

For a study to be included in this systematic review and meta-analysis it needed to be an observational study that collected information about the prevalence of *C. cayetanensis* in water. This review included studies that speciated the parasite and those that did not. Only English language studies published after 1979 (the discovery of the parasite) were eligible [5]. Prevalence cannot be calculated from case–control studies, so they were excluded from this review.

### Information sources

The databases searched included MEDLINE^®^ (Web of Science^TM^), CAB Direct (CABI), Agricola (ProQuest), Environmental Science Index (ProQuest) and Food Science and Technology Abstracts (EBESCOhost). The protocol stated that Water Resources Abstracts would be searched. However, the search was extended to include Environmental Science Index for a more expansive search. MEDLINE^®^, CAB Direct, Food Science and Technology Abstracts and Agricola were each searched on 4 August 2020, and Environmental Science Index was searched on 11 August 2020.

### Search strategy

The search was developed using terms relating to the population and outcome. To ensure an extensive search, synonyms of water along with different combinations of the parasite name were connected using ‘OR’ and the water and parasite keyword groups were linked together using ‘AND’. All databases were searched using the same strategy used for MEDLINE^®^ (Table 1). The results of this search were verified by hand-searching the reference lists of three of the most recently published relevant review articles [8, 23, 24].

**Table 1.** Search strategy used for MEDLINE^®^ (Web of Science^TM^) on 4 August 2020 to identify studies that estimated the prevalence of *Cyclospora cayetanensis* in water

#	Search string	Results
**1**	TS = (*Cyclospora cayetanensis* OR C. cayetanensis OR cyclosporiasis OR cyclospora OR C cayetanesis OR cayetanensis) Indexes = MEDLINE Timespan = 1979–2020	776
**2**	TS = (water OR sewage OR pond OR sludge OR lake OR river) Indexes = MEDLINE Timespan = 1979–2020	965, 871
**3**	1 AND 2	165

### Study selection

The results of the search were uploaded into EndNote Web and deduplicated. Then, all references were uploaded into a reference management software, DistillerSR^®^ (Evidence Partners, Ottawa, ON, Canada), where they were again deduplicated. To ensure each reference was unique, the references were exported into a Microsoft Excel spreadsheet where any remaining identical publications were manually deleted. DistillerSR^®^ was also used for the screening, data extraction and risk-of-bias assessment.

The selection process consisted of two stages: (1) title and abstract screening and (2) full-text screening. For both stages, two reviewers (TN and ST) worked independently. When the reviewers disagreed, and consensus could not be met, a third reviewer (JS) was consulted. Before starting the selection process, the screening questions were pre-tested on fifty and five references for the first and second stage, respectively.

In the first stage, only the title and abstract were used to determine eligibility. All questions required a ‘Yes’, ‘No’, or ‘Unclear’ response and a reference was only excluded if the two reviewers agreed that the response to any of the questions was ‘No’. Any article that was not excluded moved on to the second screening stage. The following questions were used for title/abstract screening:

1. Based on the title/abstract is this a study about *Cyclospora* (unspeciated or *C. cayetanensis*) and water?
2. Is this a primary research study?

The second stage of screening involved reviewing the full text using questions that required either a ‘Yes’, or ‘No’ response. If the reviewers agreed that a response to any of the questions was ‘No’, then the reference would be excluded from the review. All publications that were not excluded at this level were included in the systematic review. The following questions were used for the second stage of screening.

1. Is the full text available in English?
2. Does the full text describe a study on *Cyclospora* (unspeciated or *C. cayetanensis*) and water?
3. Does the full text describe a primary observational study (not including case report, case series, or case–control)?
4. Does the full text report the prevalence of *Cyclospora* (unspeciated or *C. cayetanensis)* or information sufficient to estimate prevalence (e.g. number of positive samples, the total number of samples)?

### Data collection process

A form was created in DistillerSR® to collect relevant data, which was expanded from that listed in the protocol to characterise the sampling and detection methods more accurately by including an additional four questions. This form was pretested by three reviewers (TN, ST and JS) on five references. Following the pretest, two reviewers (TN and ST) independently extracted data from all relevant articles using the form. Any conflicts were resolved through discussion and, if a consensus could not be met, a third reviewer was consulted. Only information provided in the article was collected and study investigators were not approached to obtain or confirm data. No assumptions were made about the data and any missing information was documented as ‘Not reported’.

### Data items

#### Study-level data

1. Time frame of data collection (months, years, seasons)
2. Location where the study was conducted (country)
3. Type of water sampled (e.g. irrigation, well, drinking)
4. Type of study (outbreak or not)

#### Outcome-Level Data

1. Prevalence of *Cyclospora*
  - Number of positive samples collected
  - Total number of samples collected
  - Reported prevalence
2. Variation
  - Type and result of variability metric (e.g. standard deviation, interquartile range)
3. Diagnostic methods
  - Water concentration methods
  - Laboratory method(s) used to identify organism
  - Whether the organism was confirmed to be *C. cayetanensis* through sequencing

### Risk of bias in individual studies

The criteria from the Risk-of-Bias Tool for Prevalence Studies developed by Hoy *et al*. for disease prevalence studies involving human subjects [25] was adapted for this review to apply to microorganisms. For example, questions addressing external validity and questions referring to study subjects were not relevant and were excluded. The following were the questions used to assess risk-of-bias:

1. Was the primary objective of the study to measure the prevalence of *C. cayetanensis* instead of an additional component of the study?
  - Yes
  - No
2. Was the same method of data collection used for all samples?
  - Yes
  - No
3. Was the same method of organism identification used for all samples?
  - Yes
  - No

Data extraction questions and risk-of-bias questions were included in the same form and two reviewers independently reviewed each article. Any disagreement between reviewers were resolved through discussion and, when consensus could not be met, a third reviewer was consulted. All information used to determine risk-of-bias was collected through the publication and no investigator was contacted to obtain or confirm information.

### Summary measures

The effect estimated for this review was prevalence. RStudio^®^ (Version 4.0.2) was used to calculate the prevalence and 95% confidence intervals from the raw data extracted from each of the studies. Adjusted prevalence was also planned to be extracted; however, the adjusted prevalence was not reported for any of the studies.

## Synthesis of results

All studies included in the systematic review were also included in the meta-analysis. Data analysis was completed in RStudio^®^ (Version 4.0.2) using the metfor, dmetar and meta packages [26–28]. A multi-level analysis was performed [29]. This analysis was not described in the protocol but was included because many studies reported more than one prevalence estimate [30]. The multi-level analysis was conducted to control for the dependence of multiple estimates from a single study. It was conducted using a random-effects model for estimating prevalence and a random effect was included for the study. Prevalence estimates extracted from each study were transformed using the Freeman –Tukey double arcsine transformation [31]. The between-study variance was estimated using the restricted maximum likelihood method. Heterogeneity was evaluated using *I*^2^ and Cochran's *Q* test. A *P*-value of less than 0.05 was used to determine significance.

As stated in the protocol [30], possible sources of statistical heterogeneity were explored through subgroup meta-analyses to determine if there was a significant difference in the mean transformed prevalence related to the following study-level variables: continent, water source, detection method, concentration method and whether the isolates were sequenced. During data extraction, the exact source of water and country from which the samples were collected was recorded. However, given the low number of studies (*N* = 33), many countries and water sources were only reported in one study. So, to allow for subgroup analyses, two variables were created. Countries were grouped by continent and the broader categories for water type were based on the source or use of the water. For example, surface freshwater included water sources such as lakes, rivers and boreholes, whereas underground water consisted of wells, deep underground water, shallow underground water, water pumps and finished piped water.

### Risk of bias across studies

This was not conducted, as the review question is descriptive.

## Results

### Study selection

**Fig. 1.**
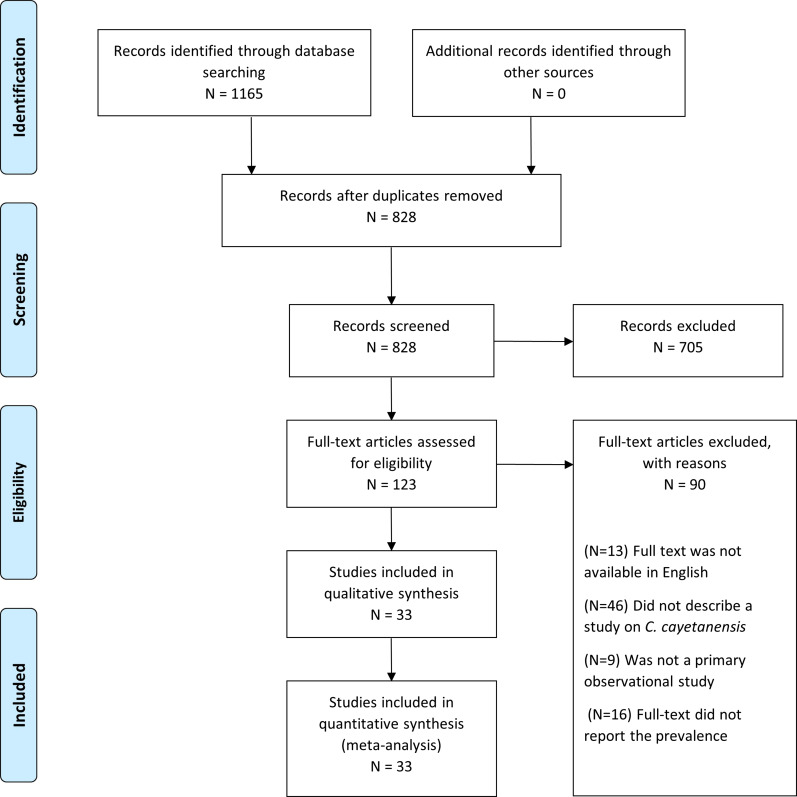
PRISMA flow diagram of studies included in the review [42].

The search generated 828 unique citations. Of these records, 705 citations were excluded based on title and abstract screening, and 90 articles were excluded based on full-text screening. Thus, 33 studies were included in the systematic review and meta-analysis (Fig. 1). All articles included in the systematic review were also included in the meta-analysis.

### Study characteristics

A summary of the characteristics of the studies included in the systematic review and meta-analysis is in Table 2. The most frequently sampled types of water were potable, tap water and river water. After categorising the types of water for subgroup meta-analyses, there were 24 prevalence estimates from drinking or household water, 19 from wastewater, 7 from irrigation water, 21 from surface freshwater, 11 from groundwater, 3 from recreational water and 7 from municipal supply water (e.g. water tanks, water treatment plant). The studies were conducted in 20 different countries, of which Egypt and Nepal were most common. When categorised into continents, there were 37 studies from Africa, 20 from Asia, 16 from North America, 12 from South America and 7 from Italy and Spain. Full details on the location, concentration method and detection method at the study level are available in Supplementary Table S1.

**Table 2.** Study characteristics from articles included in a systematic review investigating the prevalence of *Cyclospora cayetanensis* in water

Study characteristic	
Water SOurce^a^	Number of Prevalence Estimates
River	14
Drinking water	11
Tap water	11
Treated wastewater	10
Irrigation water	7
Raw wastewater	7
Well water	7
Canal	4
Lake	4
Pure ‘sachet’ water	3
Recreational	3
Water storage tanks	3
Springs	1
Borehole	2
Deep underground water (>35 m)	2
Hand washing water	2
Pond	2
Shallow underground water	2
Sludge	1
Boiled water	1
Drains	1
Finished piped water	1
Lagoon	1
Marshlands	1
Toilet water	1
‘Waterworks’^b^	1
Country	Number of Studies
Egypt	4
Nepal	4
United States	3
Colombia	2
Guatemala	2
Ghana	2
Italy	2
Zimbabwe	2
Brazil	1
Cameroon	1
China	1
Haiti	1
Malaysia	1
Nigeria	1
Peru	1
Rwanda	1
Spain	1
Tunisia	1
Turkey	1
Vietnam	1
Concentration method^c^	Number of Studies
Centrifugation	24
Filtration	16
Flocculation^d^	4
Not reported	3
Detection method^c^	Number of Studies
PCR	15
Light microscopy	12
Fluorescent microscopy	7
Confirmation of *C. cayetanensis* using sequencing	
Yes	14
No	14
No, but contains human faeces^e^	5

a Categories are not mutually exclusive, so the sum of columns does not equal the total number of prevalence estimates.

b A type of municipal supply water.

c Categories are not mutually exclusive, so the sum of columns does not equal the total number of studies.

d Flocculation is a process that converts suspended or dissolved matter into an insoluble solid which can then be removed through filtration or sedimentation.

e Identified as *Cyclospora* spp., but the water samples likely contained human faecal matter suggesting the presence of *C. cayetanensis.*

### Risk of bias within studies

For most studies (25/33), the primary objective was to measure the prevalence of *C. cayetanensis* in water. Although the majority of studies (25/33) consistently used the same method of sample collection, a smaller number of studies (6/33) did not and two studies did not report the collection method [32, 33]. Almost all of the studies (32/33) used a consistent method for the detection of *Cyclospora* spp. in each of the collected water samples; however, one study did not report whether or not the same detection method was used for all samples [33].

### Results of individual studies

Ninety-two estimates of prevalence were obtained from 33 studies. The extracted outcome data for each individual study can be found in Table S2 with details of the study characteristics.

### Results of synthesis

The summary effect from the multi-level analysis, which controlled for the dependence of estimates within studies, was 6.90% (95% CI 2.25%–13.05%, *I*^2^ = 84.38%). The Cochran's *Q* test showed that there was heterogeneity beyond that expected by chance (*P* < 0.01).

Subgroup meta-analyses were performed for location, source of water, concentration methods, detection methods and whether sequencing was performed to confirm the identity of *C. cayetanensis*. Prevalence estimates varied by region, ranging from 1.06% (CI (0.00–6.96) *I*^2^ = 68.3%) in South America to 21.06 (CI (9.26–35.45) *I*^2^ = 64.3%) in Europe. The test for subgroup differences using location as a moderator revealed a significant difference (*P* < 0.01) between locations (Table 3). Prevalence also varied between estimates that used different concentration methods such as 21.27% (CI (2.84–47.20), *I*^2^ = 78.4%) for estimates that did not report a concentration method to 3.13% (CI (0.76–6.49), *I*^2^ = 75.7%) for estimates that used centrifugation or sedimentation. The subgroup analysis using concentration methods as the moderator was significant (*P* = 0.04); however, this should be interpreted with caution since the highest prevalence came from three studies that did not report their methods and the lowest prevalence was from only one study (Table 4). To account for this, an additional subgroup analysis, with all estimates that did not report a concentration method removed (eight estimates were removed), was performed and showed that there was no significant difference between groups (Table 5). The subgroup meta-analysis for water source yielded a *P*-value > 0.05 indicating that there was no statistical difference in the prevalence estimates between the different sources of water (Table 6).

**Table 3.** Summary of the random-effects subgroup meta-analysis by continent in which water samples were collected and tested for *Cyclospora cayetanensis*

Region	Number of estimates (Number of studies)	Prevalence	95% CI (Lower; Upper)	*I*^2^ (%)
Africa	37 (12)	5.86%	(1.64; 11.63)	78.8
Asia	20 (8)	1.61%	(0.01; 4.84)	66.7
North America	16 (6)	3.46%	(0.13; 9.38)	57.5
South America	12 (4)	1.06%	(0.00; 6.96)	68.3
Europe	7 (3)	21.10%	(9.26; 35.45)	64.3

*P*-value < 0.01.

**Table 4.** Summary of the random-effects subgroup analysis on the methods used to concentrate samples of *Cyclospora cayetanensis* including studies that did not report their concentration method

Concentration method	Number of estimates	Prevalence	95% CI (Lower; Upper)	*I*^2^ (%)
Filtration	46	5.25%	(2.00; 9.47)	78.0
Centrifugation/ sedimentation	26	3.13%	(0.76; 6.49)	75.7
Flocculation followed by Centrifugation/ sedimentation	12	7.03%	(0.00; 26.13)	58.6
Not reported	8	21.27%	(2.84; 47.20)	78.4

*P*-value = 0.036.

**Table 5.** Summary of the random-effects subgroup analysis on the methods used to concentrate samples of *Cyclospora cayetanensis*

Concentration method	Number of estimates	Prevalence	95% CI (Lower; Upper)	*I*^2^ (%)
Filtration	46	5.25%	(2.00; 9.47)	78.0
Centrifugation/ sedimentation	26	3.13%	(0.76; 6.49)	75.7
Flocculation Followed by Centrifugation/ sedimentation	12	7.03%	(0.00; 26.13)	58.6

*P*-value = 0.08.

**Table 6.** Summary of the random-effects subgroup analysis on the types of water that were sampled for *Cyclospora cayetanensis*

Water source	Number of estimates	Prevalence	95% CI (Lower; Upper)	*I*^2^ (%)
Household and drinking water^a^	24	5.12%	(1.31; 10.48)	77.7
Surface freshwater^b^	21	6.61%	(2.22; 12.42)	58.8
Wastewater^c^	19	4.66%	(0.38; 11.62)	86.4
Groundwater^d^	11	0.00%	(0.00; 4.71)	53.2
Irrigation water	7	17.12%	(5.91; 31.19)	50.6
Municipal supply water^e^	7	0.18%	(0.00; 5.29)	56.2
Recreational water^f^	3	6.81%	(0.00; 34.75)	34.0

a Boiled water, ‘bowl water’ for handwashing, finished drinking water, raw drinking water, pure ‘sachet’ water, tap water.

b Borehole, canal, lake, marshlands, pond, river, runoff, springs.

c Raw wastewater, treated wastewater, sludge, drains.

d Finished piped water, deep underground water, shallow underground water, water pumps, well.

e Waterworks, water storage tanks.

f Recreational water, swimming.

*P*-value = 0.08.

Two subgroup meta-analyses were done for methods used to detect the organism (Tables 7, 8). The first analysis tested for differences between studies that used microscopy, PCR and PCR sequencing methods which resulted in an insignificant *P*-value of 0.13. The second analysis tested for differences between the different types of microscopy and PCR, because the different methods of microscopy and PCR used to identify *C. cayetanensis* have varying sensitivities and specificities [34]. This analysis yielded a significant *P*-value showing that the type of detection method contributed to heterogeneity in the prevalence estimates (Table 8).

**Table 7.** Summary of the random-effects subgroup analysis on the speciation of *Cyclospora cayetanensis* from water samples

Detection method	Number of estimates (Number of studies)	Prevalence	95% CI (Lower; Upper)	*I*^2^ (%)
Microscopy	50 (19)	3.00%	(0.0079; 0.0612 )	66.0
PCR	21 (7)	12.28%	(0.0346; 0.2407)	85.2
PCR with sequencing	21 (7)	3.68%	(0.0049; 0.0864)	77.0

*P*-value = 0.13.

**Table 8.** Summary of the random-effects subgroup analysis on the type of method used to detect *Cyclospora cayetanensis* in water samples

Detection method	Number of estimates	Prevalence	95% CI (Lower; Upper)	*I*^2^ (%)
Light microscopy	34	4.21 %	(1.42; 7.29)	70.3
Realtime PCR	16	3.69 %	(0.12; 10.05)	75.5
Standard conventional PCR	11	16.23 %	(2.33; 36.04)	69.8
Light and fluorescent microscopy	9	5.70 %	(1.04; 12.56)	40.0
Fluorescent microscopy	7	19.20 %	(0.00; 57.13)	65.8
Fluorescent microscopy and nested PCR	5	18.18 %	(0.00; 55.29)	87.0
Light microscopy and nested PCR	3	29.07 %	(6.54; 58.16)	77.2

*P*-value < 0.01.

## Discussion

In this analysis of 92 prevalence estimates from 33 studies, the pooled prevalence of *C. cayetanensis* in water was estimated to be 6.9% (95% CI (2.25–13.05), *I*^2^ = 84.34%). However, many studies reported multiple prevalence estimates, and there was substantial heterogeneity in this analysis; thus, this single value should be interpreted with caution. Although a multi-level analysis was used to control for the dependence of multiple estimates, the pooled prevalence may be biased if all the estimates from the study are biased. The results of the subgroup analyses investigating potential contributors to this heterogeneity showed that this was partially explained by continent. This was expected, since it is known that *C. cayetanensis* is endemic in tropical and subtropical regions [35]. The lowest prevalence was in South America, which was calculated to be 1.1% (95% CI (0.00–6.96), *I*^2^ = 68.3%) coming from four different studies. The small number of studies and low prevalence was surprising since South America is known to be endemic for *C. cayetanensis* [17]. Europe had the highest prevalence at 21.1% (95% CI (9.26–35.45), *I*^2^ = 64.3%), which is notable given that Europe is not an area where *C. cayetanensis* is known to be endemic. However, the seven prevalence estimates came from only three studies that took place in only two countries (Italy and Spain) and so were not representative of all of Europe. The low number of studies, particularly in areas where *C. cayetanensis* is not considered endemic is a limitation of the data. This shows that the organism exists in areas outside of subtropical regions. To fill this knowledge gap, further environmental research must be conducted in areas where *C. cayetanensis* is not endemic.

A constraint of the data is the methodologies used for detection and concentration, as they were not consistent among the 33 studies. A traditional method of detecting *C. cayetanensis* oocysts is through microscopy; however, this method lacks sensitivity and specificity [20]. PCR followed by sequence analysis is the most accurate way of detecting and distinguishing between closely related genera. In this review, only 8/33 studies used PCR and sequencing methods to detect and speciate *C. cayetanensis.* The lack of molecular detection followed by sequencing may explain the lower prevalence seen in Africa and South America, areas known to be endemic for *C. cayetanensis.* To explore this, the relationship between the region and detection was qualitatively assessed. While most estimates were from samples collected in Africa, only 8/37 used molecular methods to detect the parasite and molecular methods were most prominently used in North America (15/16 estimates) and Europe (7/7 estimates). As a result, it is possible that the organism was undetected or misidentified and the differences in detection methods used between regions may have contributed to the unexpectedly higher prevalence detected in Europe and lower prevalence in endemic areas like South America. Since *C. cayetanensis* is generally present in low concentrations in environmental samples such as water, an important step in recovering oocysts is concentrating the sample. The techniques used to concentrate the organism varied between studies and 3 studies did not report what method was used. The concentration methods used for *C. cayetanensis* are mostly techniques that were originally developed for concentrating *Cryptosporidium*. While there is limited research comparing the recovery rates of *C. cayetanensis*, there is evidence of varying efficacies among different concentration methods for recovering *Cryptosporidium* oocysts [36].

One limitation of this review was the exclusion of articles not published in English. Since *C. cayetanensis* is endemic in many countries where English is not an official language, 13 potentially relevant articles were excluded from the review. Also, there is a possibility that there is unpublished research or research without an English abstract that could not be retrieved through our search. Another limitation of the review is that we did not investigate the role of temperature or other environmental factors such as rainfall and humidity. Experiments have shown that under laboratory conditions *C. cayetanensis* can remain viable even in harsh temperatures as low as −20°C and as high as 37°C. *C. cayetanensis* has a marked seasonality and the prevalence of the organism during different seasons varies between countries [37]. Although some of the included studies took place over multiple months, there are also studies that measured the prevalence at a single point in time [38, 39]. Because of the natural seasonal variability between countries and differences in study designs, it was not possible to investigate if season or temperature had an impact on the reported prevalence.

Water scarcity is a major public health problem which impacts billions of people around the globe and is an issue that is only becoming worse due to climate change [40]. The prevalence of *C. cayetanensis* in household or drinking water was estimated to be 5.12% (95% CI (1.31–10.48), *I*^2^ = 77.7%). Since *C. cayetanensis* is transmitted through human faecal matter, the presence at any level in household or drinking water is problematic and is reflective of a person's access to clean water.

In the US between 2000 and 2017, there have been 39 foodborne outbreaks of cyclosporiasis. Since traceback investigations for foodborne illness can be difficult to conduct, only 17 of the 39 outbreaks have a confirmed or suspected source, all of which were identified as some type or combination of fresh produce [41]. While the source of the *C. cayetanensis* contamination was not identified, after considering the unique biology of the parasite and the need for oocysts to be in the environment for at least 7 days before becoming infectious, one likely route of transmission is through water used for irrigation. The subgroup analysis showed that irrigation water had the highest prevalence at 17.1% (CI (0.0591–0.3119) *I*^2^ = 50.6%), which is concerning if contaminated irrigation water is the source of food-related cases of cyclosporiasis.

Cyclosporiasis is becoming more common; however, there is limited research available on the prevalence of *C. cayetanensis* in water. A very broad search was used and yet only 33 eligible articles were identified. Even with a low number of studies, the laboratory methods used were highly variable between studies and there was an uneven distribution of studies between countries and continents. This shows that there is a need for further environmental research on *C. cayetanensis* in water using detection methods that include PCR and sequencing to accurately identify the organism.

## Data Availability

Full access to the data set used to generate the results from this review is available on request through the corresponding author.

## Supplementary material

For supplementary material accompanying this paper visit http://doi.org/10.1017/S0950268821002521. click here to view supplementary material
