# Supplementary material for: The prevalence of Cyclospora cayetanensis in water: a systematic review and meta-analysis
Source: Epidemiol Infect. 2021 Dec 10;150:e15. doi: 10.1017/S0950268821002521 (PMC8796144; doi:10.1017/S0950268821002521)
Supplement: Supplementary file 1 [file S0950268821002521sup001.docx]

Supplementary Table 1: Study characteristics for 33 studies in a systematic review and meta-analysis determining the prevalence of *Cyclospora cayetanensis* in water

| Study | Number of Samples | Location | Concentration Method | Identification Method | Use of Sequencing Methods |
| --- | --- | --- | --- | --- | --- |
| Alakpa et al 2003 [1] | 3 | Nigeria | Filtration | Light Microscopy | No |
| Ayed et al 2012 [2] | 3 | Tunisia | Centrifugation or Sedimentation | Nested PCR | Yes |
| Bern et al 1999 [3] | 1 | Guatemala | Centrifugation or Sedimentation and  Flocculation | Fluorescent Microscopy | No |
| Bilung et al 2017 [4] | 4 | Malaysia | Centrifugation or Sedimentation and  Flocculation | Light Microscopy and Fluorescent Microscopy | No |
| Dowd et al 2003 [5] | 1 | Guatemala | Centrifugation and Filtration | Standard PCR | Yes |
| El-Karamany et al 2005 [6] | 5 | Egypt | Centrifugation or Sedimentation and  Flocculation | Fluorescent Microscopy | No |
| Galvan et al 2013 [7] | 3 | Spain | Filtration | Light Microscopy and Nested PCR | Yes |
| Giangaspero et al 2015 [8] | 1 | Italy | Not Reported | RealTimePCR | Yes |
| Giangaspero et al 2015 [9] | 3 | Italy | Centrifugation or Sedimentation and  Flocculation | RealTimePCR | Yes |
| Gupta et al 2004 [10] | 1 | Nepal | Centrifugation or Sedimentation | Light Microscopy | No |
| Hoge et al 1993 [11] | 3 | Nepal | Filtration | Light Microscopy | No |
| Karaman et al 2017 [12] | 1 | Turkey | Centrifugation or Sedimentation | Light Microscopy | No |
| Khalifa et al 2001 [13] | 1 | Egypt | Centrifugation or Sedimentation | Fluorescent Microscopy | No |
| Khalifa et al 2014 [14] | 7 | Egypt | Centrifugation or Sedimentation and  Flocculation | Light Microscopy | No |
| Kitajima et al 2014 [15] | 4 | United States | Centrifugation or Sedimentation and Filtration | Real Time PCR | Yes |
| Kwakye-Nuako et al 2007 [16] | 1 | Ghana | Centrifugation or Sedimentation and Filtration | Light Microscopy | No |
| Li et al 2012 [17] | 4 | China | Centrifugation or Sedimentation | Nested PCR | Yes |
| Lopez et al 2003 [18] | 3 | Haiti | Filtration and Flocculation | Fluorescent Microscopy and Nested PCR | No |
| Masungo et al 2010 [19] | 1 | Zimbabwae | Centrifugation or Sedimentation | Light Microscopy | No |
| Mtapuri-Zinyowera et al 2014 [20] | 1 | Zimbabwae | Centrifugation or Sedimentation | Light Microscopy | No |
| Munoz-Sanchez et al 2019 [21] | 2 | Colombia | Not Reported | Standard PCR | Yes |
| Tram et al 2008 [22] | 2 | Vietnam | Centrifugation or Sedimentation and Filtration | Light Microscopy and Fluorescent Microscopy | No |
| Onstad et al 2019 [23] | 3 | United States | Centrifugation or Sedimentation and Filtration | Standard PCR | No |
| Rabold et al 1994 [24] | 1 | Nepal | Filtration | Light Microscopy | No |
| Sanchez et al 2018 [25] | 4 | Colombia | Centrifugation or Sedimentation and Filtration | Real Time PCR | Yes |
| Santos et al 2010 [26] | 4 | Brazil | Filtration | Light Microscopy | No |
| Schmitz et al 2018 [27] | 4 | United States | Centrifugation or Sedimentation and Filtration | Real Time PCR | No |
| Sherchan et al 2010 [28] | 4 | Nepal | Centrifugation or Sedimentation | Light Microscopy | No |
| Ssemanda et al 2018 [29] | 5 | Rwanda | Not Reported | Standard Conventional PCR | No |
| Sturbaum et al 1998 [30] | 2 | Peru | Centrifugation or Sedimentation and Filtration | Fluorescent Microscopy and Nested PCR | No |
| Youssef et al 1998 [31] | 3 | Egypt | Centrifugation or Sedimentation | Light Microscopy | No |
| Nsoh et al 2016 [32] | 4 | Cameroon | Centrifugation or Sedimentation | Light Microscopy | No |
| Tetteh-Quarcoo et al 2016 [33] | 3 | Ghana | Centrifugation or Sedimentation | Light Microscopy | No |

Supplementary Table 2: Results of individual studies of 92 prevalence estimates from 33 studies in a systematic review and meta-analysis examining the prevalence of *Cyclospora cayetanensis* in water

| Study | Type of Water Examined | Prevalence | Confidence Interval |
| --- | --- | --- | --- |
| Alakpa et al 2003 [1] | Pure “sachet” water | 0.00 | [0.0000; 0.1194] |
|  | Irrigation water from well | 0.067 | [0.00; 0.32] |
|  | Tap water | 0.00 | [0.00; 0.41] |
| Ayed et al 2012 [2] | Raw wastewater | 0.01 | [0.00; 0.05] |
|  | Treated wastewater | 0.00 | [0.00; 0.03] |
|  | Sludge | 0.00 | [0.00; 0.26] |
| Bern et al 1999 [3] | River | 0.07 | [0.00; 0.22] |
| Bilung et al 2017 [4] | Raw drinking water treatment plant | 0.17 | [0.02; 0.48] |
|  | Raw drinking water treatment plant | 0.00 | [0.00; 0.26] |
|  | Lake | 0.33 | [0.04; 0.78] |
|  | River | 0.00 | [0.00; 0.46] |
| Dowd et al 2003 [5] | Community drinking water | 0.25 | [0.05; 0.57] |
| El-Karamany et al 2005 [6] | Drains | 1.00 | [0.03; 1.00] |
|  | irrigation canals  shallow underground water | 1.00 | [0.03; 1.00] |
|  | Shallow underground water | 1.00 | [0.03; 1.00] |
|  | finishshed pipe water | 1.00 | [0.03; 1.00] |
|  | deep underground water | 0.00 | [0.00; 0.98] |
| Galvan et al 2013 [7] | drinking water treatment plan | 0.25 | [0.07; 0.52] |
|  | waste water treatment plant | 0.54 | [0.34; 0.72] |
|  | river basins | 0.08 | [0.00; 0.38] |
| Giangaspero et al 2015 [8] | toilet on trains | 0.30 | [0.07; 0.65] |
| Giangaspero et al 2015 [9] | well water | 0.06 | [0.00; 0.30] |
|  | treated irrigation water | 0.21 | [0.14; 0.31] |
|  | drinking water | 0.00 | [0.00; 0.71] |
| Gupta et al 2004 [10] | River | 0.00 | [0.00; 0.34] |
| Hoge et al 1993 [11] | Tap water | 0.05 | [0.00; 0.23] |
|  | water storage tanks | 0.00 | [0.00; 0.15] |
|  | drinking water | 0.00 | [0.00; 0.15] |
| Karaman et al 2017 [12] | River | 0.06 | [0.03; 0.12] |
| Khalifa et al 200 [13] | household water tanks | 0.09 | [0.04; 0.16] |
| Khalifa et al 2014 [14] | River | 0.06 | [0.01; 0.17] |
|  | Waterworks | 0.06 | [0.01; 0.17] |
|  | Tap water | 0.00 | [0.00; 0.07] |
|  | Water pumps | 0.02 | [0.00; 0.11] |
|  | Water tanks | 0.08 | [0.02; 0.20] |
|  | Pond | 0.10 | [0.03; 0.23] |
|  | Canal | 0.08 | [0.02; 0.20] |
| Kitajima et al 2014 [15] | 1- Treatment Plant (untreated water) | 0.25 | [0.05; 0.57] |
|  | 1- Treatment Plant (treated water) | 0.08 | [0.00; 0.38] |
|  | 2- Treatment Plant (untreated water) | 0.25 | [0.05; 0.57] |
|  | 2- Treatment Plant (treated water) | 0.17 | [0.02; 0.48] |
| Kwakye-Nuako et al 2007 [16] | Drinking Water | 0.59 | [0.39; 0.78] |
| Li et al 2012 [17] | 1- Wastewater treatment plant | 0.00 | [0.00; 0.04] |
|  | 2- Wastewater treatment plant | 0.06 | [0.02; 0.13] |
|  | 3- Wastewater treatment plant | 0.06 | [0.02; 0.12] |
|  | 4- Wastewater treatment plant | 0.00 | [0.00; 0.04] |
| Lopez et al 2003 [18] | 1 - Well | 0.11 | [0.00; 0.48] |
|  | 2 - Well | 0.00 | [0.00; 0.19] |
|  | 3 - Well | 0.00 | [0.00; 0.11] |
| Masungo et al 2010 [19] | Tap water | 0.00 | [0.00; 0.46] |
| Mtapuri--Zinyowera et al 2014 [20] | Wells, tap water, and boreholes | 0.10 | [0.02; 0.27] |
| Munoz-Sanchez et al 2019 [21] | Boiled water | 0.00 | [0.00; 0.12] |
|  | Tap water | 0.00 | [0.00; 0.31] |
| Tram et al 2008 [22] | Tap water | 0.13 | [0.05; 0.25] |
|  | irrigation water | 0.13 | [0.09; 0.26] |
| Onstad et al 2019 [23] | 1- River | 0.25 | [0.01; 0.81] |
|  | 2- River | 0.00 | [0.00; 0.71] |
|  | 3- River | 0.00 | [0.00; 0.60] |
| Rabold et al 1994 (24) | Water storage tanks | 1.00 | [0.03; 1.00] |
| Sanchez et al 2018 [25] | 1- Drinking water treatment plant | 0.00 | [0.00; 0.15] |
|  | 2- Drinking water treatment plant | 0.00 | [0.00; 0.15] |
|  | 3- Drinking water treatment plant | 0.00 | [0.00; 0.15] |
|  | 4- Drinking water treatment plant | 0.00 | [0.00; 0.08] |
| Santos et al 2010 [26] | 1- River | 0.00 | [0.00; 0.14] |
|  | 2- River | 0.00 | [0.00; 0.26] |
|  | 1- Lake | 0.00 | [0.00; 0.26] |
|  | 2- Lake | 0.00 | [0.00; 0.26] |
| Schmitz et al 2018 [27] | 1- Treatment Plant (untreated water) | 0.00 | [0.00; 0.26] |
|  | 1- Treatment Plant (treated water) | 0.00 | [0.00; 0.10] |
|  | 2- Treatment Plant (untreated water) | 0.00 | [0.00; 0.26] |
|  | 1- Treatment Plant (treated water) | 0.00 | [0.00; 0.06] |
| Sherchan et al 2010 [28] | Tap water | 0.00 | [0.00; 0.10] |
|  | Ponds | 0.08 | [0.00; 0.38] |
|  | Well | 0.00 | [0.00; 0.11] |
|  | Canal | 0.25 | [0.03; 0.65] |
| Ssemanda et al 2018 [29] | River | 0.17 | [0.00; 0.64] |
|  | Marshland | 0.17 | [0.00; 0.64] |
|  | Lake run-off | 0.67 | [0.22; 0.96] |
|  | Lakes | 0.67 | [0.22; 0.96] |
|  | Ground water | 0.33 | [0.04; 0.78] |
| Sturbaum et al 1998 [30] | Lagoon | 1.00 | [0.29; 1.00] |
|  | Wastewater | 0.50 | [0.16; 0.84] |
| Youssef et al 1998 [31] | Water tank | 0.08 | [0.00; 0.38] |
|  | Canal | 0.00 | [0.00; 0.46] |
|  | Swimming pools | 0.00 | [0.00; 0.60] |
| Nsoh et al 2016 [32] | Borehole | 0.40 | [0.19; 0.64] |
|  | Springs | 0.00 | [0.00; 0.41] |
|  | Tap water | 0.18 | [0.11; 0.25] |
|  | Well water | 0.13 | [0.00; 0.53] |
| Tetteh-Quarcoo et al 2016 [33] | School water | 0.00 | [0.00; 0.19] |
|  | Water before hand washing | 0.11 | [0.01; 0.35] |
|  | Water after hand washing | 0.11 | [0.01; 0.35] |

1. **Alakpa GE, Clarke SC, Fagbenro-Beyioku AF**. *Cyclospora cayetanensis* infection: vegetables and water as possible vehicles for its transmission in Lagos, Nigeria. *British Journal of Biomedical Science* 2003; **60**: 113–114.

2. **Ben Ayed L, *et al.*** Survey and genetic characterization of wastewater in Tunisia for  *Cryptosporidium spp.* ,  *Giardia duodenalis* ,  *Enterocytozoon bieneusi* , *Cyclospora cayetanensis*  and  *Eimeria spp.* . *Journal of Water and Health* 2012; **10**: 431–444.

3. **Bern C, *et al.*** Epidemiologic studies of *Cyclospora cayetanensis* in Guatemala. *Emerging Infectious Diseases* 1999; **5**: 766–774.

4. **Bilung LM, *et al.*** Detection of *Cryptosporidium* and *Cyclospora* Oocysts from Environmental Water for Drinking and Recreational Activities in Sarawak, Malaysia. *BioMed Research International* Hindawi, 2017; **2017**: e4636420.

5. **Dowd SE, *et al.*** Confirmed detection of *Cyclospora cayetanesis*, *Encephalitozoon intestinalis* and *Cryptosporidium parvum* in water used for drinking. *Journal of Water and Health* 2003; **1**: 117–123.

6. **El-Karamany EMN, Zaher TI, El-Bahnasawy MM**. Role of water in the transmission of cyclosporiarsis in Sharkia Governorate, Egypt. *Journal of the Egyptian Society of Parasitology* 2005; **35**: 953–962.

7. **Galván AL, *et al.*** Molecular Characterization of Human-Pathogenic *Microsporidia* and *Cyclospora cayetanensis* Isolated from Various Water Sources in Spain: a Year-Long Longitudinal Study. *Applied and Environmental Microbiology* American Society for Microbiology, 2013; **79**: 449–459.

8. **Giangaspero A, Marangi M, Arace E**. *Cyclospora cayetanensis* travels in tap water on Italian trains. *Journal of Water and Health* 2015; **13**: 210–216.

9. **Giangaspero A, *et al.*** Molecular detection of *Cyclospora* in water, soil, vegetables and humans in southern Italy signals a need for improved monitoring by health authorities. *International Journal of Food Microbiology* 2015; **211**: 95–100.

10. **Gupta R, *et al.*** Study on *Cyclospora cayetanensis* in Kathmandu valley. *JNMA, Journal of the Nepal Medical Association* 2004; **43**: 258–263.

11. **Hoge CW, Shlim DR, Rajah R (et al.)**. Epidemiology of diarrhoeal illness associated with coccidian-like organism among travellers and foreign residents in Nepal. *Lancet* 1993; **341**: 1175–1179.

12. **Karaman U, *et al.*** The Protozoa and helminths in the water of Terme and Kocaman boroughs of Samsun province. *Turgut Özal Tıp Merkezi Dergisi* 2017; **24**: 472–476.

13. **Khalifa AM, El-Temsahy MM, El-Naga IFA**. Effect of ozone on the viability of some protozoa in drinking water. *Journal of the Egyptian Society of Parasitology* 2001; **31**: 603–616.

14. **Khalifa RMA, *et al.*** Present status of protozoan pathogens causing water-borne disease in northern part of El-Minia Governorate, Egypt. *Journal of the Egyptian Society of Parasitology* 2014; **44**: 559–566.

15. **Kitajima M, *et al.*** Occurrence of *Cryptosporidium*, *Giardia*, and *Cyclospora* in influent and effluent water at wastewater treatment plants in Arizona. *Science of the Total Environment* 2014; **484**: 129–136.

16. **Kwakye-Nuako G, *et al.*** Sachet drinking water in Accra: the potential threats of transmission of enteric pathogenic protozoan organisms. *Ghana Medical Journal* 2007; **41**: 62–67.

17. **Li Na, *et al.*** Molecular surveillance of *Cryptosporidium spp., Giardia duodenalis* , and  *Enterocytozoon bieneusi*  by genotyping and subtyping parasites in wastewater. *PLoS Neglected Tropical Diseases* 2012; **6**: e1809.

18. **Lopez AS, *et al.*** Epidemiology of *Cyclospora cayetanensis* and other intestinal parasites in a community in Haiti. *Journal of Clinical Microbiology* 2003; **41**: 2047–2054.

19. **Masungo P, Dube T, Makaka C**. A survey of the diversity of human enteric protoctistan parasites and the associated risk factors in urban Zvishavane, Zimbabwe. *Agriculture and Biology Journal of North America* 2010; **1**: 985–991.

20. **Mtapuri-Zinyowera S, *et al.*** Human parasitic protozoa in drinking water sources in rural Zimbabwe and their link to HIV infection. *Germs* 2014; **4**: 86–91.

21. **Muñoz-Sánchez GD, *et al.*** Food protozoa safety assessment and risk in school restaurants in Armenia, Colombia. *Journal of Food Safety* 2019; **39**: e12714.

22. **Tram NT, *et al.*** *Cyclospora spp.* in herbs and water samples collected from markets and farms in Hanoi, Vietnam. *Tropical Medicine & International Health* 2008; **13**: 1415–1420.

23. **Onstad NH, *et al.*** *Cyclospora cayetanensis* Presence in the Environment—A Case Study in the Chicago Metropolitan Area. *Environments* 2019; **6**Published online: 1 January 2019.doi:10.3390/environments6070080.

24. **Rabold JG, Hoge CW, Shlim DR**. *Cyclospora* outbreak associated with chlorinated drinking water. *in foreigners in Nepal* 1994; **344**: 1360–1361.

25. **Sánchez C, *et al.*** Molecular detection and genotyping of pathogenic protozoan parasites in raw and treated water samples from southwest Colombia. *Parasites and Vectors* 2018; **11**: (26 October 2018).

26. **Santos SFO, *et al.*** Environmental monitoring of opportunistic protozoa in rivers and lakes in the neotropics based on yearly monitoring. *Water Quality, Exposure and Health* 2010; **2**: 97–104.

27. **Schmitz BW, *et al.*** Reduction of *Cryptosporidium*, *Giardia*, and fecal indicators by Bardenpho wastewater treatment. *Environmental Science & Technology* 2018; **52**: 7015–7023.

28. **Sherchan JB, *et al.*** Infection of *Cyclospora cayetanensis* in diarrhoeal children of Nepal. *Journal of Nepal Paediatric Society* 2010; **30**: 23–30.

29. **Ssemanda JN, *et al.*** Foodborne pathogens and their risk exposure factors associated with farm vegetables in Rwanda. *Food Control* 2018; **89**: 86–96.

30. **Sturbaum GD, *et al.*** Detection of *Cyclospora cayetanensis* in wastewater. *Applied and Environmental Microbiology* 1998; **64**: 2284–2286.

31. **Youssef MYM, Khalifa AM, El-Azzouni MZ**. Detection of *Cryptosporidia* in different water sources in Alexandria by monoclonal antibody test and modified Ziehl Neelsen stain. *Journal of the Egyptian Society of Parasitology* 1998; **28**: 487–496.

32. **Nsoh FA, *et al.*** Prevalence, characteristics and correlates of enteric pathogenic protozoa in drinking water sources in Molyko and Bomaka, Cameroon: a cross-sectional study. *BMC Microbiology* 2016; **16**: 268.

33. **Tetteh-Quarcoo PB, Anim-Baidoo I, Kwaku Attah S**. *Microbial Content of “Bowl Water” Used for Communal Handwashing in Preschools within Accra Metropolis, Ghana*. (https://www.hindawi.com/journals/ijmicro/2016/2617473/). Accessed 7 July 2021.
